# Has Alberta Oil Sands Development Altered Delivery of Polycyclic Aromatic Compounds to the Peace-Athabasca Delta?

**DOI:** 10.1371/journal.pone.0046089

**Published:** 2012-09-26

**Authors:** Roland I. Hall, Brent B. Wolfe, Johan A. Wiklund, Thomas W. D. Edwards, Andrea J. Farwell, D. George Dixon

**Affiliations:** 1 Department of Biology, University of Waterloo, Waterloo, Ontario, Canada; 2 Department of Geography and Environmental Studies, Wilfrid Laurier University, Waterloo, Ontario, Canada; 3 Department of Earth & Environmental Sciences, University of Waterloo, Waterloo, Ontario, Canada; Utrecht University, The Netherlands

## Abstract

**Background:**

The extent to which Alberta oil sands mining and upgrading operations have enhanced delivery of bitumen-derived contaminants via the Athabasca River and atmosphere to the Peace-Athabasca Delta (200 km to the north) is a pivotal question that has generated national and international concern. Accounts of rare health disorders in residents of Fort Chipewyan and deformed fish in downstream ecosystems provided impetus for several recent expert-panel assessments regarding the societal and environmental consequences of this multi-billion-dollar industry. Deciphering relative contributions of natural versus industrial processes on downstream supply of polycyclic aromatic compounds (PACs) has been identified as a critical knowledge gap. But, this remains a formidable scientific challenge because loading from natural processes remains unknown. And, industrial activity occurs in the same locations as the natural bitumen deposits, which potentially confounds contemporary upstream-downstream comparisons of contaminant levels.

**Methods/Principal Findings:**

Based on analyses of lake sediment cores, we provide evidence that the Athabasca Delta has been a natural repository of PACs carried by the Athabasca River for at least the past two centuries. We detect no measureable increase in the concentration and proportion of river-transported bitumen-associated indicator PACs in sediments deposited in a flood-prone lake since onset of oil sands development. Results also reveal no evidence that industrial activity has contributed measurably to sedimentary concentration of PACs supplied by atmospheric transport.

**Conclusions/Significance:**

Findings suggest that natural erosion of exposed bitumen in banks of the Athabasca River and its tributaries is a major process delivering PACs to the Athabasca Delta, and the spring freshet is a key period for contaminant mobilization and transport. This baseline environmental information is essential for informed management of natural resources and human-health concerns by provincial and federal regulatory agencies and industry, and for designing effective long-term monitoring programs for the lower Athabasca River watershed.

## Introduction

Industrial development of oil sands deposits in northern Alberta, Canada, has grown rapidly during the past few decades. Production is projected to reach >3.3 million barrels per day by 2020 [1] and generate $300 billion in tax revenue over the next 25 years [2]. The economic and oil-security benefits are undeniable, but public concerns continue to grow over the potential downstream cumulative environmental effects of the oil sands industry on the lower Athabasca River watershed and residents of its communities. Debate has become polarized in response to the accelerating pace of development. This has led to numerous recent reports by expert review panels that conclude current monitoring programs are inadequate to address the growing concerns [3]–[5].

**Figure 1:**
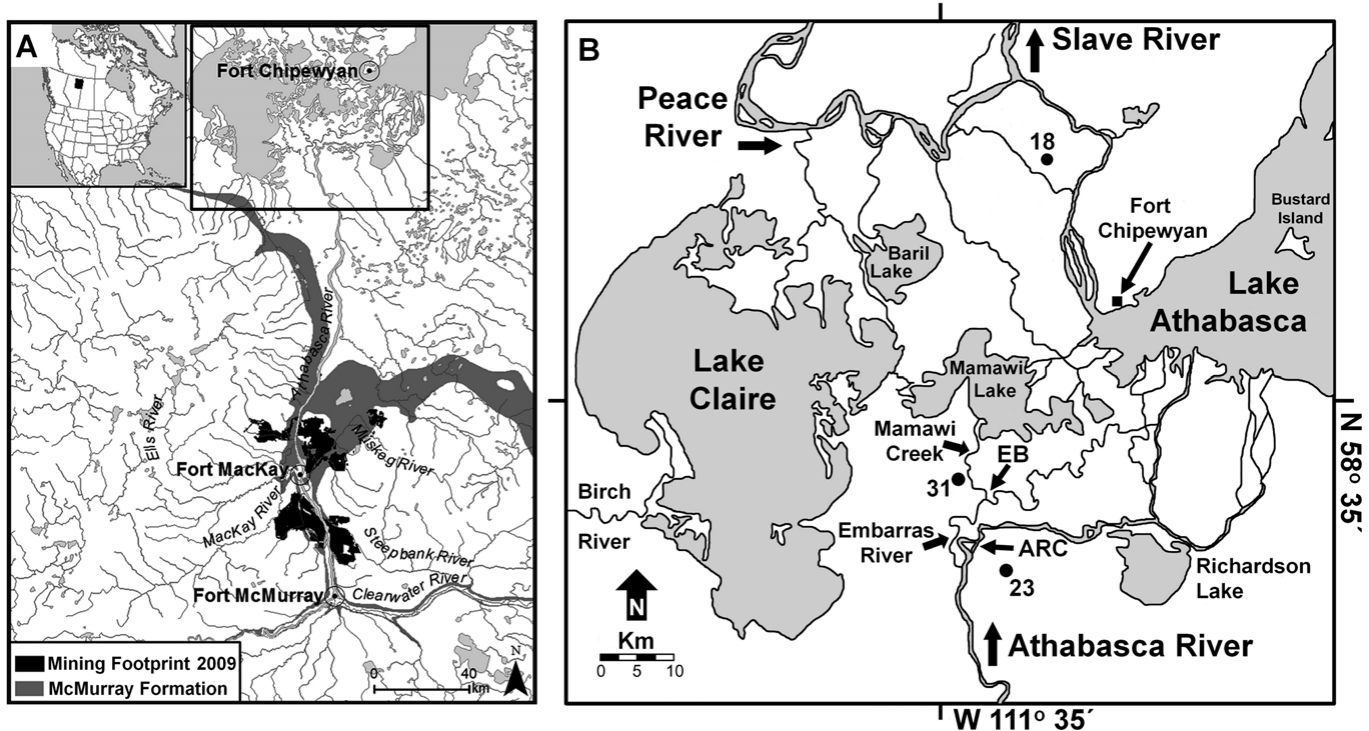
Maps of study area. Panel a): map showing locations of near-surface Athabasca oil sands exposures of the McMurray Formation (light grey), the 2009 mining footprint (dark grey) and the downstream Peace-Athabasca Delta (top center box) in northern Alberta, Canada. Top-center box in **a)** outlines area shown in Panel **b)**: Map identifying locations of the study lakes (solid circles) PAD 18 (N 58° 53.7′, W 111° 21.7′), PAD 23 (N 58° 23.5′, W 111° 26.6′) and PAD 31 (N 58° 29.6′, W 111° 31.0′) within the Peace-Athabasca Delta. Also shown are locations of the Athabasca River Cutoff (ARC) and the Embarras Breakthrough (EB). The ARC, an engineered channel excavation in 1972, straightened and deepened the Athabasca River at the location of a large meander bend where ice-jam floods occurred, and reduced flood frequency at PAD 23. The EB, a natural avulsion that occurred in 1982, has increased flood frequency at PAD 31 because it directs substantial and increasing Athabasca River flow towards PAD 31 via Mamawi Creek. Further details on these geomorphic changes are provided in [27].

Focal points of concern include the downstream community of Fort Chipewyan, the lower Athabasca River, Lake Athabasca and the Peace-Athabasca Delta (PAD; Fig. 1) where worries have been expressed regarding effects of increasing oil sands development on human and ecosystem health [1], [6], [7]. There, First Nation and Métis residents value a traditional way of life, and many believe that contaminants emitted from the oil sands industry are responsible for higher-than-expected rates of diseases including cancers [e.g., 6] and perceived increases of fish deformities [7]. Of particular concern are polycyclic aromatic compounds (PACs), because many PACs in bitumen are known or suspected human carcinogens [8] and have been associated with toxicological effects in fish [9]. Recent studies have identified that concentrations of some PACs and metals are elevated in snowpack and river water within 50 km of the oil sands development, and concentrations of some metals remain greater in river water at the PAD than upstream of development [10], [11]. Yet, other lines of evidence point to the delta as a natural repository for contaminants. For instance, in Lake Athabasca and Richardson Lake, which continuously receive inflow from the Athabasca River, PAC concentrations are high and comparable in sediments deposited pre- and post-development [12]. Black, bitumen-rich sediment in strata deposited during the early 1800s in oxbow lakes of the PAD prone to floods from the Peace River, which passes through unexploited surficial deposits of oil sands, has also been reported [13]. These findings provide evidence that natural fluvial processes deposit contaminants downstream in lakes of the PAD, but relative contributions of contaminants from natural versus industrial causes remain unknown.

Discriminating industrial contributions from natural loads is difficult, and it remains a critical scientific challenge because the Athabasca River and its tributaries traverse surface deposits of the bitumen-rich McMurray Formation in the same areas where surface mining and processing occur. As a consequence, natural sources confound attempts to attribute differences between upstream and downstream levels of contaminants to industrial activity [3]. Moreover, PACs are a diverse group of organic compounds created by natural processes as well as by human activities [8], [14].

To date, monitoring programs have been unable to establish baseline reference conditions of natural contaminant loading to the delta, a situation identified as “almost unique in monitoring for toxic compounds” by the Federal Expert Oil Sands Advisory Panel [3]. Based on attempts to analyze PAC data of the Regional Aquatics Monitoring Program (RAMP) from annual collections of bulk river sediment, Timoney and Lee [15] recently suggested that downstream PAC concentrations increased during 1999–2009, which they concluded “may be related to industrial activities” [p. 4282 in 15] based on correlations between PAC concentrations and particulate emissions and bitumen production by oil sands development. However, these results are based on periodic collection of samples of the upper 4–6 cm of river sediment. As acknowledged by the authors, uncertainty regarding the time interval represented by these samples is substantial, with individual samples potentially encompassing time periods that approach the entire decade of sample collection. These data are further complicated by the possibility of sediment remobilization and loss given the dynamic nature of fluvial and fluvio-deltaic depositional environments [16]. In fact, the sampling approach has been identified as problematic [1], [3], [17], in part because these factors are likely sufficient to mask any meaningful temporal trends. Thus, the suggestion that industrial activity has caused measureable enhancement of pollutant fluxes to the delta remains a testable hypothesis, rather than a scientific certainty.

Unfortunately, RAMP monitoring and other scientific studies began several decades after industrial development of the oil sands, so they cannot readily identify nor distinguish any additional contaminant loading due to industry beyond what is contributed by natural processes [3]. And, they have not adequately characterized contaminant transport during the spring freshet, an erosive annual event when natural contaminant dispersal occurs in northern rivers because ice-scour is substantial and flow velocity is relatively high [18], [19]. This is when accumulated industrial-sourced contaminants in the winter snowpack become released as a pulse to the rivers, but its importance for dispersal of industrial contaminants remains a key unknown [1], [7], [10], [11]. Releases of PACs during the spring melt are of particular concern because they coincide with fish spawning [1], [20] and when river flow conditions maximize dispersal [19]. Clearly, *w*ithout such knowledge, concerns about contaminant delivery to the delta will inevitably increase as oil sands development expands.

Here, we utilize analyses of precisely-dated lake sediment cores to establish baseline, pre-development, reference conditions for PAC deposition in the PAD. This represents the only method available to directly measure PAC concentrations prior to industrial development of the oil sands, as required to characterize the role of natural riverbank erosion as a vector for downstream deposition of PACs. Indeed, it is an approach recommended by the Federal Expert Oil Sands Advisory Panel [3] and advocated by Environment Canada’s Phase 2 monitoring program [21]. We assess whether the Athabasca River and its tributaries have been important vectors for PAC accumulation in the downstream Athabasca Delta over the past ∼200 years. We then address the question: Is there evidence to indicate that industry has measurably altered the composition and concentration of PACs in lake sediments in the Athabasca Delta from that provided by natural supplies due to riverbank erosion and subsequent downstream transport by the Athabasca River? We also use our findings to test the hypothesis [10] that oil sands development has altered sedimentary composition and concentration of PACs due to regional atmospheric transport to the delta.

## Methods

### Ethics Statement

Permission for collection of the sediment samples was provided by Parks Canada Agency (Wood Buffalo National Park) Research and Collection Permit WB-2010-6893.

### Study Design

**Figure 2:**
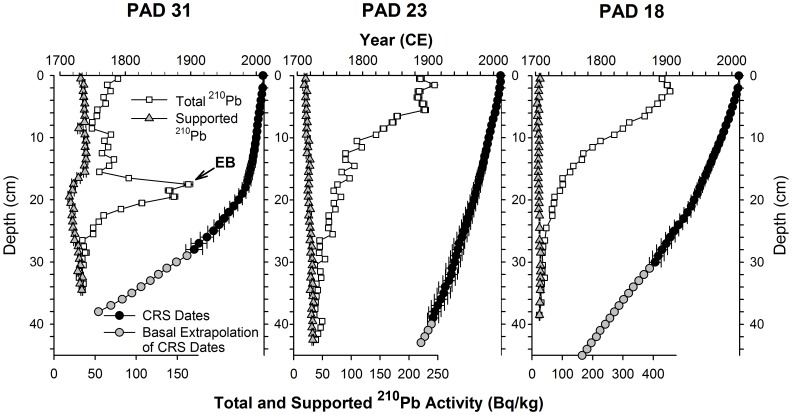
Sediment core dating. ^210^Pb activity (supported and total ( = supported+unsupported)) and depth-age profiles for sediment cores taken from the three study lakes (PAD 31, PAD 23, PAD18) in the Peace-Athabasca Delta, northern Alberta (error bars = ±2 SD). The irregular ^210^Pb activity profile for PAD 31 is a result of an increase in sedimentation rate associated with increased flooding following the Embarras Breakthrough in 1982 [27].

**Figure 3:**
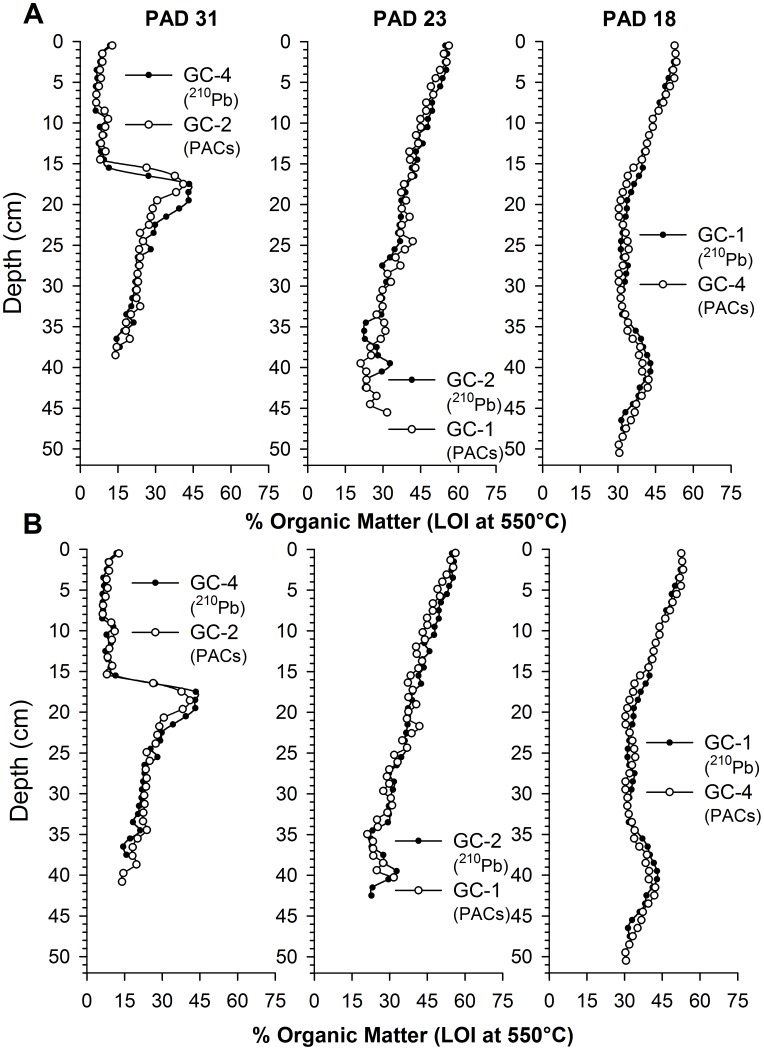
Alignment of duplicate cores. Graphs showing stratigraphic profiles of organic matter content in duplicate cores taken from each of the three study lakes in the Peace-Athabasca Delta, northern Alberta. One of the cores from each lake was analyzed for ^210^Pb dating and the other provided material for analysis of PACs. Upper panel **a)** shows the raw data, whereas the lower panel **b)** shows the data after minor vertical adjustment of the organic matter content profile for the core that was not dated. ^210^Pb-derived dates from the dated core were transferred to the corresponding undated cores used for PAC analyses. No depth adjustments were required for cores from PAD 18.

Our study design capitalizes on knowledge of past hydrological conditions that we have gained from more than a decade of field-based studies in the PAD, which is essential for informed interpretation of stratigraphic records of PACs given the hydrological complexity of the delta over space and time [22]–[32]. Three previously studied and strategically-positioned lakes were selected for stratigraphic analyses of PACs (Fig. 1). Lakes ‘PAD 23’ and ‘PAD 31’ (unofficial names) are located in the Athabasca Delta, but have markedly different flood histories during the latter part of the 20th century [27]. PAD 23 (elevation = 213.3 m a.s.l.), one of the most elevated lakes within the Athabasca Delta, was prone to flooding until the engineered Athabasca River Cutoff of 1972 allowed flow to bypass a bend in the river where ice-jams had previously formed and routed floodwaters towards PAD 23 [27]. Thus, sediments deposited before 1972 were designated as flood-prone, whereas sediments deposited afterwards were designated as not flood-prone. The pre-1972 flood-prone period was further divided into two periods, pre- and post-1933. Sediments deposited pre-1933 were characterized by lower organic matter (OM) content, lower cellulose-inferred lake water δ^18^O values and markedly lower content of macrofossils from the submerged moss *Drepanocladus* compared to post-1933 sediments, suggesting stronger river influence pre-1933 [22]. In contrast, PAD 31 (elevation ∼209.5 m a.s.l.), which lies approximately 40 cm above nearby Mamawi Lake (an open-drainage lake receiving continuous river inflow ),has experienced three distinct periods of differing hydrological conditions during the past >200 years. PAD 31 was designated as flood-prone from the base of the core (pre-1800) until 1940 when it was frequently flooded and inundated under a former highstand of Lake Athabasca, as identified by low sediment OM content and abundant open-drainage indicator diatom taxa [22], [30]. During 1940 to 1982, PAD 31 was not flood-prone and existed as an isolated closed-drainage lake due to declining Lake Athabasca water levels, as identified by declining relative abundance of open-drainage indicator diatom taxa and a shift to dominance by taxa indicative of closed-drainage conditions in previously analyzed cores from PAD 31, as also occurred in other delta lakes around 1940 (see Johnston et al.’s Fig. 7 [ref. 30]). PAD 31 became flood-prone for a second time after the 1982 Embarras Breakthrough event, a natural avulsion that diverted substantial flow from the Athabasca and Embarras rivers into Cree and Mamawi creeks and towards PAD 31, as identified by a visible contact between darker organic-rich sediment pre-Breakthrough and lighter mineral-rich sediments afterwards, a marked rise in sedimentation rate (Fig. 2), a sharp decline in OM content (Fig. 3) and changes in other sedimentary variables [27]. At present, PAD 31 is one of the most flood-prone of the restricted-drainage lakes in the Athabasca Delta [22], [27]. With knowledge of these site-specific hydrological changes, analyses of PACs in lake sediment cores from PAD 23 and PAD 31 provide data to identify the role of the Athabasca River as a source of contaminant loading to the PAD. In contrast, ‘PAD 18’, an elevated (214.3 m a.s.l.) closed-drainage lake located in the northern Peace Delta, has not flooded for at least the past 100 years [22]. Consequently, all sediment samples were defined as not flood-prone. To assess influence of Athabasca oil sands exploration on atmospheric deposition of PACs, the PAD 18 sediment record was divided into pre- and post-1967 onset of oil sands mining activity [1], [4]. Also, we obtained two samples of flood-deposited river sediment from the ground surface at two locations between Mamawi Creek and PAD 31 (approximately mid-way and 50 m from PAD 31) shortly after an ice-jam flood event along the Athabasca River in spring of 2007. These sediments, hereafter referred to as the ‘2007 Athabasca Delta flood deposit’, provided reference materials to assess the composition and concentration of PACs supplied to PAD lakes by Athabasca River floodwaters.

### Field Sampling and Laboratory Analyses

In September 2010, four sediment cores, 27–53 cm in length, were retrieved from each of PAD 18, 23, 31 using a gravity corer [33] and transported to the field base in Fort Chipewyan where they were sectioned into 1-cm stratigraphic intervals. Samples were kept refrigerated (4°C) in the dark prior to lab analyses. Sequential loss-on-ignition (LOI) analyses were performed on ∼0.5 g subsamples of wet sediment from all core slices to provide profiles of water (post 90°C for 24 hours), OM (post 550°C for 1 hour) and carbonate (post 950°C for 1 hour) contents, following standard methods [34]. The OM content profiles were used to transfer the sediment core chronology developed from a single dated core per lake to a second core that was analyzed for the composition and concentration of PACs (Fig. 2, 3).

### ^210^Pb Dating of Sediment Cores

One sediment core per lake was selected for dating by gamma ray spectrometric determination of ^210^Pb activity at contiguous 1-cm intervals. A measured mass of ∼2-4 g of dry sediment was packed into pre-weighed tubes (SARSTEDT product No. 55.524) to a uniform height of 35 mm and a TFA Silicone Septa (Supelco®) was placed overtop the sediment, followed by 1 cm^3^ of 2 Ton Clear Epoxy (Devcon® product No. 14310). Three weeks were allowed for ^222^Rn and its decay products to reach equilibrium with ^226^Ra (parent isotope) prior to quantifying ^210^Pb, ^214^Bi and ^214^Pb activity. Activities of the radioisotopes were measured in an Ortec co-axial HPGe Digital Gamma Ray Spectrometer (Ortec GWL-120-15) interfaced with Maestro 32 software (version 5.32) at the University of Waterloo’s WATER Lab. Sample count time ranged from 23–95 hours per sample, and varied so that net ^210^Pb counts were >10 × the standard deviation of the net counts of a well-defined machine blank to ensure precision [35]. Calibration of detector energy efficiency for the radioisotopes was previously established for our gamma ray spectrometers using a similarly prepared known standard (3.012 g of IAEA 300, see [36]). Detection efficiency values were determined from the mean of three sample runs performed using the IAEA-300 standard. ^210^Pb activities were decay-corrected to the coring date for each lake and the ^210^Pb activity was density-corrected as described by [37].

The Constant Rate of Supply (CRS) model [38], [39] was used to develop the sediment core chronologies. The CRS model requires an estimation of supported ^210^Pb activity, which was determined from activity of ^214^Bi and ^214^Pb. Results showed that supported ^210^Pb activity is variable over time in the sediment cores (Fig. 2). To reduce influence of this temporal variability, a five-point running mean of ^214^Pb and ^214^Bi activity was used as an estimate of the supported ^210^Pb activity for a given interval. The core depth where total ^210^Pb activity equals supported ^210^Pb activity was determined using standard methods [40].

The sediment chronology for the ^210^Pb-dated core was transferred to the core analyzed for PACs based on alignment of OM content profiles in both cores determined by LOI analysis (Fig. 3). Slight depth offsets in the OM content profiles of cores from PAD 23 and PAD 31 were adjusted using a constant depth multiplier (+6% and −11.5%, respectively, for PAD 31 GC-2 and PAD 23 GC-1). This approach preserved sample-to-sample variability in the sedimentation rate determined for the dated core. The CRS chronology was then applied to the modified depth interval of the core analyzed for PACs by a straight linear interpolation of the nearest-neighbour dates (depth-wise) of the ^210^Pb-dated core (e.g., if 10 and 11 cm-depth in the ^210^Pb-dated core corresponded to 2000 and 1998.5 respectively, then a modified sample depth of 10.6 cm depth in the core analyzed for PACs equates to 1999.1). Results are consistent with cores we have previously analyzed for these lakes [22], [27]. Also, the stratigraphic contact at 18 cm depth marking the 1982 Embarras Breakthrough was used to confirm alignment of cores from PAD 31.

### PAC Analyses

Samples from one core from each of the study lakes were analyzed for PACs at ALS Canada Ltd. (Edmonton). Using knowledge of the water content derived from LOI analysis, an amount of wet sediment equivalent to ≥2 g dry sediment (mean = 4.6 g dry mass) was placed into amber glass jars with Teflon lid-liners that were pre-washed with acid (5% HCl) and rinsed with solvent (dichloromethane). We combined adjacent sediment intervals for the uppermost water-rich samples in cores from PAD 18 and PAD 23 to obtain sufficient sample mass. Additionally, two samples of the 2007 Athabasca Delta flood deposit were analyzed. These flood deposit samples were kept refrigerated since collection and were similarly prepared and shipped with the other sediment core samples to ALS for analysis of 52 PACs and alkylated PACs using US Environmental Protection Agency method EPA 3540/8270-GC/MS.

Identification and quantification of individual PACs had good repeatability based on analyses of replicate subsamples (mean precision: ±12.5% of PAC concentration averaged across all replicate analyses). Where repeated measurements of a sample were available, the average value was used for data analysis and display. In a few instances, an individual PAC was below detection limit (e.g., <0.040 mg/kg) in one replicate but slightly above the detection limit (e.g., 0.042 mg/kg) in the other replicate for a given sediment interval (e.g., FLPY2 in the sample at 35–36 cm from PAD 18). In these cases, a random number between zero and the detection limit was generated for the first replicate below the detection limit, and then averaged with the value obtained for the second replicate to avoid underweighting the PAC concentration. For situations where all measurements for a given PAC in a sediment interval were below the analytical detection limit, the PAC measurement was treated as having a concentration of zero.

### Numerical Analyses

A one-way Analysis of Similarities (ANOSIM) test, a nonparametric and multivariate approximate analogue of analysis of variance (ANOVA) tests [41], was performed on the relative abundances (expressed as proportions of the PAC sum) of the quantified PACs in all sediment core samples from the three study lakes (n = 127) to determine if composition of PACs differs between sediments deposited during flood-prone versus not flood-prone intervals, based on assignment of stratigraphic intervals as described above. ANOSIM tests are commonly, and increasingly, applied to determine differences in biological community composition among site-categories [41], and they are now being used for analysis of contaminant composition [42]. It is common practice to remove rarely encountered species from multivariate analysis and transform the data to up-weight or down-weight uncommon species, depending upon research goals [41]. We opted not to remove rare PACs for this study to avoid the possibility of dismissing historically-rare compounds that could have become recently deposited in the PAD due to anthropogenic activities or other processes. Our intent was to analyze the full PAC dataset with as little manipulation as possible. Consequently, the data were not transformed prior to calculating Bray-Curtis similarity coefficients used by ANOSIM computations. The 2007 Athabasca Delta flood deposit samples were not used in the ANOSIM test or the SIMPER analysis described below. Instead, they served as an independent benchmark to help identify river-transported PACs in the stratigraphic records. The test statistic (global R) computed by the ANOSIM test reflects the observed differences between samples in the two categories (flood-prone versus not flood-prone) contrasted with the differences among replicates within each group, and it ranges from 0 to 1. A value of 0 indicates that the similarity between and within groups is the same on average. A value of 1 indicates that replicates within a group are more similar to each other than to all other replicates of other groups [41]. A p-value was computed by comparing the distribution of within- and across-group rank Bray-Curtis similarities (999,999 computations) to the initial rank similarity, as reported by the global R value [41], [43]. For the ANOSIM test, we set alpha = 0.05.

A one-way Similarity of Percentages (SIMPER; [43]) analysis was performed on the full, untransformed relative abundance PAC data to identify which of the 43 detected PACs best discriminated between samples deposited during flood-prone versus not flood-prone intervals. As detailed below, this provided a method to select PACs indicative of a bitumen origin and transport by Athabasca River floodwaters versus those that are more abundant due to other processes. ANOSIM and SIMPER analyses were performed using the software PRIMER, version 6.1.5 [41], [43].

For PAD 31, the study lake that was flood-prone for several decades both before and after onset of oil sands development, independent-samples t-tests were performed to determine if the mean of the sum of the river-transported bitumen-associated indicator PACs, identified by use of SIMPER analysis, differed in sediments deposited during flood-prone periods pre- versus post-onset of oil sands development. Independent-samples t-tests were performed on these data when expressed as concentrations and as proportions, because Kolmogorov-Smirnov tests identified that the distributions do not differ significantly from normal (P>0.05). Non-parametric Mann-Whitney tests provided the same conclusions as the independent-samples t-tests. These tests were performed using the software SPSS version 16.0.

## Results

### PAC Composition

**Figure 4:**
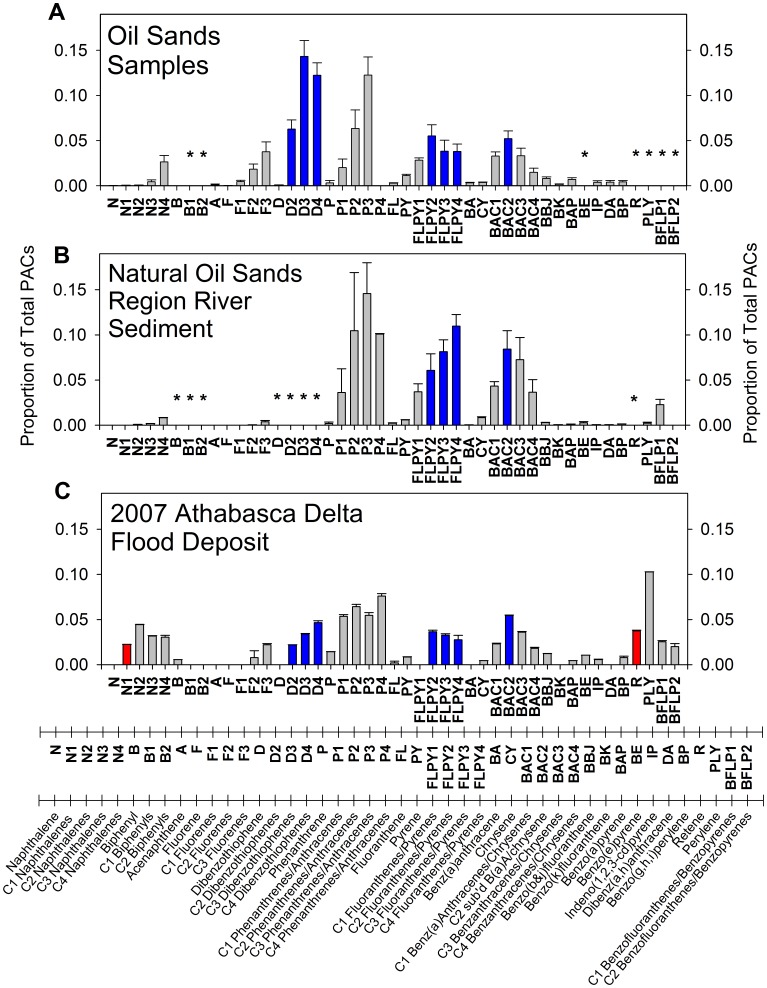
Composition of polycyclic aromatic compounds in oil sands and river sediment downstream of McMurray Formation. Bar graphs showing average composition of polycyclic aromatic compounds (PACs), expressed as relative concentration (proportion; error bars = ±1 SEM) in **a)** oil sands samples from Syncrude, Steepbank River mouth, and east and west banks of the Athabasca River (from [10]), **b)** natural oil sands region river sediment (from [44]) and **c)** the 2007 Athabasca Delta flood deposit. Names of the PAC compounds and the corresponding codes are presented along the lower expanded horizontal axis. Blue vertical bars are PACs identified by SIMPER analysis as river-transported bitumen-associated indicator PACs deposited during flood-prone intervals (Table 1). Red vertical bars are PACs identified as common in sediments deposited during not flood-prone periods. * identifies PACs that were not measured in one sample but were measured and reported in another sample presented in the figure.

The composition of PACs in the 2007 Athabasca Delta flood deposit (Fig. 4c) closely matches ‘reference’ compositions reported for oil sands samples within the oil sands region (Fig. 4a; [10]), and for sediments in the Athabasca River and two tributaries downstream of riverbank deposits of bitumen in areas unaffected by industrial development (Fig. 4b; [44]). The oil sands samples and the natural oil sands region river sediments reported by [10], [44] originate from sites located ∼200 km upstream of the Athabasca Delta. Based on geochemical fingerprinting, the main source of the PACs in the natural oil sands region river sediments is bitumen-rich material in the riverbanks [45]. Similar to the oil sands samples and the reference river sediments, the 2007 Athabasca Delta flood deposit was dominated by alkyl-substituted PACs, including C4 naphthalenes (N4), C2- through C3-fluorenes (F2–F3), C2- through C4-dibenzothiophenes (D2–D4), C1- through C4-phenanthrenes/anthracenes (P1–P4), C2- through C4-fluoranthenes/pyrenes (FLPY2-FLPY4) and C1- through C4-benzo[a]anthracenes/chrysenes (BAC1–BAC4) (Fig. 4). Compounds C1- through C3-naphthalenes (N1–N3) were also common in the 2007 Athabasca Delta flood deposit. Levels of D2–D4 in the 2007 Athabasca Delta flood deposit could not be compared with the natural bitumen-rich Athabasca River sediments because dibenzothiophenes were not analyzed by [44] (Fig. 4b), but they are a major component of the oil sands samples (Fig. 4a; [10]). Except for perylene, unsubstituted (parent) PACs were absent or rare in the 2007 Athabasca Delta flood deposit. Perylene is formed naturally by metabolic decomposition of plant matter by bacteria and fungi [46], [47] and is associated with transport of terrestrial materials from watersheds and with in-situ generation in aquatic systems [47]–[49], suggesting that the Athabasca River also transports PACs of terrestrial vegetation origin to the Athabasca Delta. However, the predominance of alkyl-substituted PACs over unsubstituted PACs in both the 2007 Athabasca Delta flood deposit and reference oil sands samples and river sediments (Fig. 4; [10], [44], [45]) identify that a major component of the sediment-associated PACs transported downstream and to the Athabasca Delta by the Athabasca River during floods derives from a common petrogenic origin – namely, bitumen from exposures of the McMurray Formation along the riverbanks in the oil sands area.

**Table 1:** Indicator PACs for flood-prone and not flood-prone sediments.

	Between-group Dissimilarity (Flood-prone *vs* Not flood-prone)		Within-group Similarity (Flood-prone or Not Flood-prone)		Average Proportion of total PACs (0–1)	
	Average Dissimilarity %	Contribution %	Average Similarity %	Contribution %	Flood-prone	Not flood-prone
Indicator PACs for Flood-prone intervals						
C4 Dibenzothiophenes	2.30	4.53	2.25	3.10	0.05	0.00
C3 Dibenzothiophenes	2.07	4.08	3.42	4.71	0.04	0.00
C2 sub’d B(a)A/chrysene	1.93	3.79	3.03	4.18	0.04	0.00
C2 Fluoranthenes/Pyrenes	1.25	2.46	1.73	2.38	0.03	0.00
C2 Dibenzothiophenes	1.21	2.39	1.59	2.19	0.02	0.00
C3 Fluoranthenes/Pyrenes	1.14	2.25	1.58	2.18	0.02	0.00
C4 Fluoranthenes/Pyrenes	0.97	1.92	1.09	1.50	0.02	0.00
Indicator PACs for Not flood-prone intervals						
Naphthalene	8.47	16.65	14.25	23.90	0.05	0.21
C1 Naphthalenes	4.86	9.56	9.00	15.09	0.03	0.13
Retene	1.92	3.78	4.14	6.95	0.03	0.06

Results of similarity percentages (SIMPER) analysis of PAC composition in sediment core samples from the three study lakes of the Peace-Athabasca Delta, northern Alberta, with samples categorized as flood-prone or not-flood prone, to identify PACs most indicative of river transport versus other vectors and processes.

**Figure 5:**
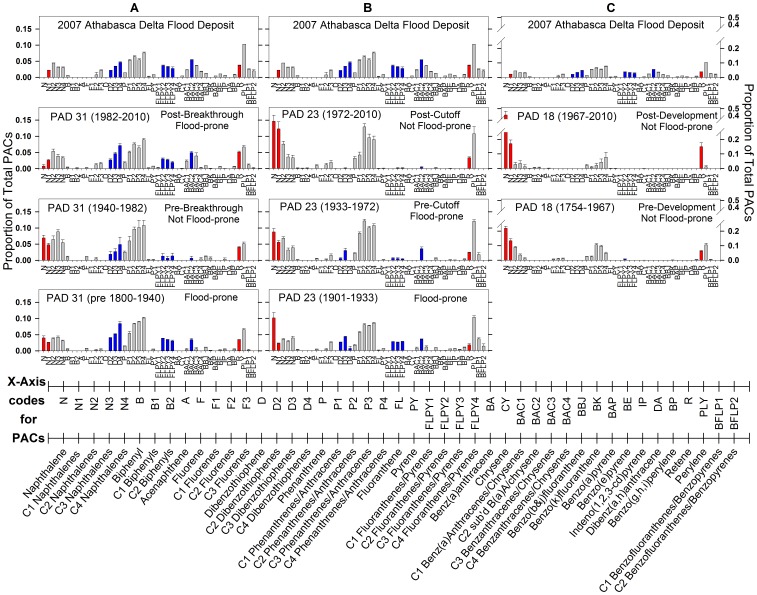
Composition of polycyclic aromatic compounds in river flood sediment and lake sediment cores. Bar graphs showing average composition of polycyclic aromatic compounds (PACs), expressed as relative concentration (proportion; error bars ±1 SEM) in **a)** the 2007 Athabasca Delta flood deposit and three intervals of differing flood regime at PAD 31, **b)** the 2007 Athabasca Delta flood deposit and three intervals of differing flood regime at PAD 23, and **c)** the 2007 Athabasca Delta flood deposit and two intervals that distinguish pre- and post-1967 onset of major commercial oil sands development at not flood-prone PAD 18 (note different scale of vertical axis). Names of the PAC compounds and the corresponding codes are presented along the lower expanded horizontal axis. Blue vertical bars are PACs identified by SIMPER analysis as river-transported bitumen-associated indicator compounds common in sediments deposited during flood-prone intervals (Table 1). Red vertical bars are PACs identified as indicator compounds in sediments deposited during not flood-prone periods.

The average composition of PACs in sediments deposited in PAD 31 during the two periods when the lake was flood-prone (pre-1940 and post-1982) closely matches that of the 2007 Athabasca Delta flood deposit (Fig. 5a, panels 1, 2, 4), and to that of bitumen-rich natural deposits located upstream (see Fig. 4) [10], [44], [50]. PACs in these sediments contain high proportions of the alkyl-substituted forms C2- through C4-naphthalenes (N2–N4), C2- through C3-fluorenes (F2–F3), C2- through C4-dibenzothiophenes (D2–D4), C1- through C4-phenanthrenes/anthracenes (P1–P4), C2- through C4-fluoranthenes/pyrenes (FLPY2–FLPY4) and C1- through C4-benzo[a]anthracenes/chrysenes (BAC1–BAC4). Other relatively abundant PACs are retene (R) and perylene (PLY). Retene, a C4-phenanthrene, is associated with combustion of plant material [51], [52]. Only minor differences occur between sediments deposited in PAD 31 when it was flood-prone and the 2007 Athabasca Delta flood deposit. Specifically, naphthalene (N) is absent in the 2007 Athabasca Delta flood deposit, but is present in low proportions in PAD 31 sediments deposited post-1982 (<1%) and pre-1940 (<4%). Also, only subtle differences occur in PAD 31 sediments deposited during the two flood-prone periods, with slightly lower proportions of F2–F3, BAC1–BAC3 and R, and a slightly higher proportion of N in sediments deposited pre-1940 compared to post-1982. Clearly, the sediment record from PAD 31 identifies that bitumen-derived PACs carried by the Athabasca River are deposited in this lake when it has received river floodwaters, and that this has occurred both before and since onset of industrial development of the oil sands.

By contrast, the average composition of PACs in sediments from PAD 31 when the lake was not flood-prone (1940–1982: Fig. 5a, panel 3) differed notably from that of the 2007 Athabasca Delta flood deposit, as well as sediments deposited in PAD 31 during the two flood-prone periods (pre-1940, post-1982). Specifically, sedimentary proportions of F3, D2–D4, FLPY2–FLPY4 and BAC1–BAC3 were markedly lower when PAD 31 was not flood-prone (1940–1982), and proportions of N, C1-naphthalenes (N−1), biphenyl (B) and fluorene (F) were more than double. Overall, the data show that the composition of PACs in PAD 31 sediments differs detectably between periods of frequent versus infrequent flooding. During flood-prone periods, PAC composition closely matches that of river-transported sediment originating from bitumen deposits of the McMurray Formation exposed along the riverbanks (Fig. 4, 5). During periods of reduced flood frequency, higher proportions of unsubstituted PACs (notably N, B, F) identify greater influence of hydrocarbons from fire and catchment vegetation [46], [52], [53].

At PAD 23, the average composition of PACs in sediments deposited post-Cutoff (post-1972), when flood frequency at the lake dropped precipitously [27], is very different from that of the 2007 Athabasca Delta flood deposit (Fig. 5b, panels 1, 2). Many of the alkylated PACs abundant in the flood deposit (D2–D4, FLPY2–FLPY4, BAC1–BAC4) were nearly absent in post-Cutoff sediments at PAD 23. Also, N and N−1 dominated the PACs in post-Cutoff sediments at PAD 23, but these compounds are absent or in low relative abundance, respectively, in the flood deposit. The high proportions of unsubstituted N, F and PLY, and R in PAD 23 sediments during this ‘not flood-prone’ period suggest the PACs originate from a pyrogenic source (e.g., forest fires) as well as biodegradation products from plant materials [47], [50], [51], [53].

Composition of PACs in sediments deposited at PAD 23 prior to the Cutoff (1972), when the lake was prone to flooding from the Athabasca River [27], is notably different compared to the post-Cutoff period of reduced flood frequency. During the pre-Cutoff flood-prone period, N, N1 and F were relatively less abundant on average, and several of the alkylated PACs common in the 2007 Athabasca Delta flood deposit (D2–D3, FLPY2–FLPY4, BAC2) were more abundant (Fig. 5b, panels 3, 4). Interestingly, this pattern was more strongly apparent during the earliest portion of the flood-prone period (1901–1933), when paleohydrological data identify greater influence of flooding at PAD 23 [27]. Also, PAC composition during the flood-prone periods at PAD 23 shows some similarities with the subsequent period of reduced flood frequency (post-Cutoff), with generally higher relative abundance of N, N1 and P2–P4 than in the 2007 Athabasca Delta flood deposit. This suggests a mixture of PACs from both petrogenic sources supplied by river floodwaters and pyrogenic sources, likely due to the lake’s proximity to forests subject to periodic fires.

Based on data obtained previously, PAD 18 has not received floodwaters for at least the past century [22]. The composition of PACs in sediments from this lake, during both pre- and post-oil sands development periods, is markedly different than that of the 2007 Athabasca Delta flood deposit, the natural oil sands region river sediments and sediments deposited during flood-prone periods in the other two study lakes (Fig. 4; Fig. 5c, panels 2, 3). PACs in sediments from PAD 18 are overwhelmingly dominated by N, N1 and R. And, the alkylated PACs characteristic of river-transported bitumen in the 2007 Athabasca Delta flood deposit and during flood-prone periods at PAD 31 and PAD 23 (notably, F2–F3, D2–D4, FLPY3–FLPY4, BAC1–BAC3) are absent at PAD 18. Only FLPY2 was detected, but at low relative abundance (3–5%) and only in five samples deposited pre-1900. Thus, PACs in sediments at PAD 18 are characteristic of mainly pyrogenic and vegetation sources, with little to no inputs from PACs of petrogenic origin.

### Identification of River-transported Bitumen-associated Indicator PACs

Based on results of a one-way analysis of similarities (ANOSIM) test, performed on the relative abundance of the PACs in sediment core samples from the three study lakes (Fig. 5), PAC composition differs significantly between flood-prone versus not flood-prone periods in the study lakes (R-statistic = 0.499, P<1.0×10^−6^, n = 127). This finding confirms a strong influence of river floodwaters on the sedimentary composition of PACs.

Subsequent similarities percentages (SIMPER) analysis was used to identify the ‘indicator’ PAC compounds that most strongly contribute to differences in PAC composition between the flood-prone and not flood-prone periods of the study lakes. Ten of the 43 PACs were found to have good indicator qualities: seven for flood-prone sediment intervals (D2–D4, FLPY2–FLPY4, BAC2) and three for not flood-prone sediment intervals (N, N1, R; Table 1). Collectively, these ten PACs accounted for >50% of the total dissimilarity between samples deposited during flood-prone and not flood-prone intervals.

We sought to identify compounds that were consistently more abundant in either the flood-prone or not flood-prone sediment samples and that strongly contribute to the within-group similarity of one sample-type (but not both). This selection criterion was met for the PACs indicative of flood-prone intervals. The seven PAC indicators of flood-prone status (D2–D4, FLPY2–FLPY4, BAC2) had higher relative abundances (proportionally >2x) in sediments deposited during flood-prone intervals and contributed consistently to the between-group (flood-prone versus not flood-prone) dissimilarity (dissimilarity/std. dev. >1, contribute >1% to dissimilarity; Table 1). Individually, they contributed substantially to the within-group similarity of flood-prone samples (>1%), but not to the within-group similarity of the not flood-prone samples (<0.1%). These ‘river-transported indicator PACs’ (coloured blue in Fig. 4, 5) contributed 21.42% to the total between-group dissimilarity for flood-prone versus not flood-prone samples, and 20.24% to the total within-group similarity of the flood-prone samples (Table 1). They contributed only 0.11% to the total within-group similarity of not flood-prone samples. They averaged 22% of the total PACs present in sediments in the flood-prone samples, but were <1% of the total PACs present in the not flood-prone samples. These river-transported indicator PACs, based on our independent statistical analysis, are in agreement with PACs previously identified as characteristic of bitumen-rich materials associated with the oil sands of the McMurray Formation (Fig. 4) [10], [44], [45], [50]. In fact, these seven river-transported indicator PACs account for 51% of the total PACs in oil sands samples reported by Kelly *et al*. [10]. Thus, we refer to them hereafter as ‘river-transported bitumen-associated PACs’.

Using the same criteria, SIMPER analysis identified three PACs indicative of sediments deposited during not flood-prone intervals (N, N1, R; Table 1). These three indicator PACs were consistently found at higher relative abundances in sediments deposited during the not flood-prone intervals (proportionally >2x), contributed consistently to the between-group (flood-prone versus not flood-prone) dissimilarity (dissimilarity/std. dev. ≥0.8), and contributed considerably more to the within-group similarity of the not flood-prone samples than that of the flood-prone samples. These ‘not flood-prone indicator PACs’ (coloured red in Fig. 4, 5) contributed 29.90% to the total dissimilarity between flood-prone and not flood prone-samples and 45.94% to the total within-group similarity of the not flood-prone samples. They contributed 10.51% to the total within-group similarity of flood-prone samples. On average, they comprised 40% of the total PACs found in the not flood-prone samples compared with 11% for flood-prone samples. The indicator PACs for sediments deposited during not flood-prone periods are rare or absent in oil sands bitumen [10], [44], [54] and are often associated with a pyrogenic source such as forest fires [14], [51], [52]. However, they also occurred in sediments deposited during flood-prone intervals, suggesting that these PACs are most strongly associated with delivery to lakes at the Athabasca Delta by non-river sources and vectors, such as atmospheric transport of PACs produced by fires and from biological degradation of vegetation within the catchment. But, river floodwaters may also acquire these compounds from terrestrial landscapes and deliver them downstream to the delta [55].

Phenanthrene/Anthracene homologues (P1–P4) were not identified by SIMPER analysis as indicator PACs because they were common in all sediment samples (Fig. 4, 5). Indeed, P1–P4 are common in river sediments both upstream and downstream of Athabasca oil sands deposits [44], as well as in river sediments draining unburned and burned forests of northern Alberta in areas outside the Athabasca oil sands region [52].

### Relations between River-transported Bitumen-associated Indicator PAC Concentration and Sediment Organic Matter Content

**Figure 6:**
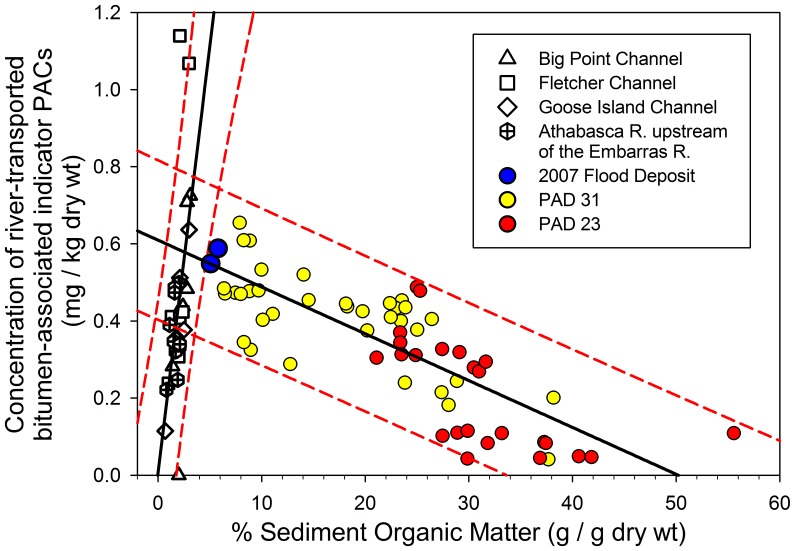
Relations between polycyclic aromatic compound concentration and organic matter content in river versus lake sediment. Scatterplot showing the relations between organic matter (as a percentage of dry sediment mass) and concentration of river-transported bitumen-associated indicator PACs in sediments of lakes PAD 23 (red circles) and PAD 31 (yellow circles) in the Athabasca Delta (data from this study), in bottom sediments of the Athabasca River and its distributaries at four stations within the Athabasca Delta (open symbols; data from [56]), and in sediments deposited on land between Mamawi Creek and PAD 31 by an ice-jam flood in spring of 2007 (blue circles; data from this study). Data include the sum of the concentration of six of the seven river-transported bitumen-associated indicator PACs that were common to both the lake sediment data set (this study) and the river bottom sediment from RAMP [56] (note: FLPY-4 was not reported by [56]). The lines of best fit (solid black line) and 95% prediction intervals (dashed red lines) are presented for linear regressions of sediment organic matter content and river-transported bitumen-associated indicator PAC concentrations for both the river bottom sediments and the lake sediments.

Two distinct trends are evident when comparing river-transported bitumen-associated indicator PAC concentrations in river and lake sediments in the Athabasca Delta versus organic matter content, which further reveal processes responsible for deposition of these PACs (Fig. 6). Data obtained from RAMP [56] show that the organic content of river-bottom sediments is much lower than that of the lake sediments. Also, concentrations of the river-transported bitumen-associated indicator PACs in river sediment are positively correlated with organic matter content of bottom sediments obtained in the Athabasca River and distributaries within the delta (R^2^ = 0.35, P = 0.0004, d.f. = 30; Fig. 6). This positive association probably derives mainly from variations in grain size distribution and composition of river sediment, because the finer-grained fraction possesses higher organic matter content and higher affinity for hydrophobic contaminants, such as PACs [57], than coarser fractions.

In contrast to the sediments of the Athabasca River and distributaries, river-transported bitumen-associated indicator PAC concentrations in the lake sediment records of PAD 23 and PAD 31 are significantly and *negatively* correlated with organic matter content (R^2^ = 0.63, P<0.0001, d.f. = 60; Fig. 6). This relation reflects periodic inoculation of the lakes with river-borne sediments that are relatively low in organic matter content, but relatively high in river-transported bitumen-associated PAC concentration. Under non-flood conditions, concentrations of the river-transported bitumen-associated PACs in the lake sediments are diluted by in-lake aquatic productivity that increases organic matter content. A similar pattern has been reported for sediments of floodplain lakes in the Mackenzie Delta [55]. The presence of higher concentrations of river-transported bitumen-associated PACs in PAD 31 (and correspondingly lower organic matter content) compared to PAD 23 is consistent with the more proximal location of PAD 31 to river floodwaters. Notably, high river-transported bitumen-associated PAC concentrations in the 2007 flood deposits obtained near PAD 31 lie close to the intersection of the two relations, confirming that flood events deposit river sediment and associated river-transported bitumen-associated PACs directly into PAD 31.

### River-transported Bitumen-associated Indicator PAC Profiles in Sediment Cores

**Figure 7:**
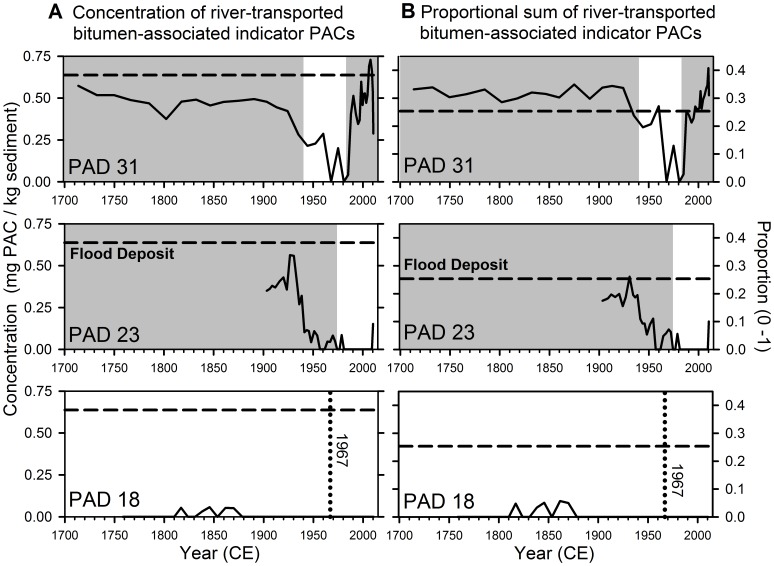
Time trends of river-transported bitumen-associated indicator PACs. Stratigraphic records from sediment cores of the three study lakes (PAD 31, 23, 18) showing **a)** the sum of the concentrations of the seven river-transported bitumen-associated indicator PACs (C2-through C4-dibenzothiophenes, C2- through C4-fluoranthenes/pyrenes, C2-sub’d B(a)A/chrysene), and **b)** the sum of the proportions of the seven river-transported bitumen-associated indicator PACs. Grey shading identifies periods when the lakes were flood-prone. Absence of shading identifies periods when the lakes were not flood-prone. The dotted vertical line in plots for PAD 18 identifies 1967, the year when major commercial operations began [1], [4]. Dashed horizontal lines in panels **a)** and **b)** correspond to the average value for the sum of the seven river-transported bitumen-associated indicator PACs in the 2007 Athabasca Delta flood deposit, where **a)** shows the concentration ( = 0.638 mg/kg) and **b)** shows the proportion ( = 0.254).

Summing the seven PACs identified by the SIMPER analysis as indicative of flood-prone conditions provided an approach to quantify changes over time in the proportion and concentration of bitumen-associated, river-transported PACs to the three study lakes, as described below (Fig. 7).

At PAD 31, the average concentration of the river-transported bitumen-associated indicator PACs does not differ significantly between sediments deposited since the Embarras Breakthrough in 1982 (0.476 mg/kg ±0.165 (1 SD)), when the lake became frequently and increasingly flooded by channelized flow of Athabasca River-sourced water along Mamawi Creek, and sediments deposited pre-1940 when PAD 31 received sediments from the Athabasca River when frequently submerged under a highstand of Lake Athabasca (0.466 mg/kg ±0.065 (1 SD); independent-samples t-test, P = 0.821; d.f. = 31; Fig. 7a, top panel). The independent-samples t-test achieved greater than 99% power to detect a mean difference of 0.1 mg/kg (∼1 SD), indicating low probability of a Type-2 error. Indeed, concentrations of the summed river-transported bitumen-associated indicator PACs fell close to that of the 2007 Athabasca Delta flood deposit during flood-prone periods both prior to and since onset of oil sands development. Peak concentration occurred in 2007, the year when ice-jam flooding delivered the reference Athabasca Delta flood deposit to the lakeshore. The two other concentration peaks post-1982 corresponded with high open-water flow on the Athabasca River (1990) and ice-jam floods (1996–97; [58], [59]). Consistent with this evidence of strong regulation of sediment PACs by river flooding, the sum of the river-transported bitumen-associated indicator PACs (expressed as both concentration and proportion) declined to very low levels during 1940–1982 when PAD 31 was not flood-prone (except for two peaks within this period that correspond to ice-jam flood years of 1963–64 and 1974 [58]). Interestingly, the average proportion of river-transported bitumen-associated indicator PACs during 1983–2010 (27.1% ±7.9 (1 SD)) was significantly lower than during the pre-1940 interval (31.5% ±2.8 (1 SD); independent-samples t-test, P = 0.044, d.f. = 31; Fig. 7b, top panel). Overall, these data provide evidence that the proportion and concentration of accumulated river-transported bitumen-associated indicator PACs have not increased measurably at PAD 31 since onset of oil sands development.

At PAD 23, highest amounts of river-transported bitumen-associated indicator PACs were deposited in sediments before ∼1940 (middle panels in Fig. 7a,b), when prior research shows strongest flooding [27]. The long-term 20^th^ century decline in proportions and concentrations of river-transported bitumen-associated indicator PACs likely reflects declining susceptibility of this basin to floods [27]. Except for a brief rise in the sample that includes 1974, the year of a known major ice-jam flood event [58], and in the top-most sample, possibly representing a recent unrecorded flood event, river-transported bitumen-associated indicator PACs have been virtually absent in sediments deposited in PAD 23 since the Athabasca River Cutoff (1972). These findings highlight that natural erosion and transport via the Athabasca River are major processes delivering these contaminants.

At PAD 18, where floods have been absent for the past century [22], river-transported bitumen-associated indicator PACs were not detected, except for very low concentrations (0.032–0.057 mg/kg) of FLPY2 during the early- to mid-1800s (bottom graphs in Fig. 7a,b). Clearly, the river-transported bitumen-associated indicator PACs are not supplied in substantial amounts to lakes in the absence of flooding, but their presence in a few samples suggests that PAD 18 may have received a few floods during this time, which was marked by notably high water levels in Lake Athabasca [30].

### Total PAC Concentration Profiles in Sediment Cores

**Figure 8:**
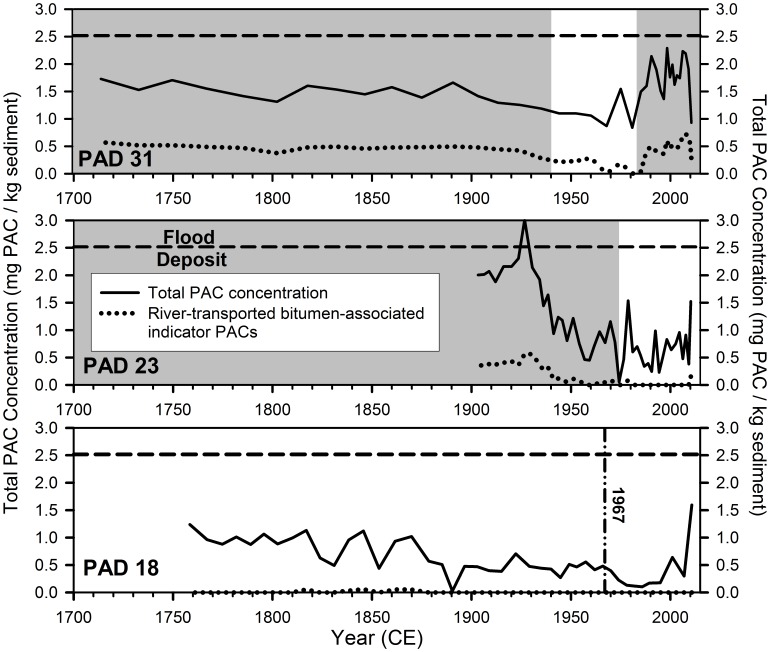
Time trends of total PAC concentration. Stratigraphic records showing the total PAC concentration (solid line) and the concentration of the seven PACs indicative of a bitumen origin and transport by the Athabasca River (dotted line) in the sediment cores from lakes PAD 31, PAD 23 and PAD 18. Grey shading identifies periods when the lakes were flood-prone. Absence of shading identifies periods when the lakes were not flood-prone. The vertical dashed-dotted line in the plot for PAD 18 identifies 1967, the year when major commercial oil sands operations began [1], [4]. The dashed horizontal line in all three panels corresponds to the average value for the total PAC concentration in the 2007 Athabasca Delta flood deposits (2.518 mg/kg).

Total sedimentary PAC concentrations ranged from 0.053 to 3.008 mg/kg at PAD 23, from 0.837 to 2.234 mg/kg at PAD 31 and from 0.0271 to 1.596 mg/kg at PAD 18 (Fig. 8). Similar to the seven river-transported bitumen-associated indicator PACs, concentrations of total PACs are markedly higher during the flood-prone versus not flood-prone periods (Fig. 8). Analyses of sediment cores from PAD 31 and PAD 23 identified transient increases in total and river-transported bitumen-associated indicator PAC concentrations in samples dating to 1974, a year of widespread flooding that inundated both lakes despite their not flood-prone status. At PAD 31, three distinct peaks occur in total PAC concentration during the post-1982 flood-prone period (∼1990, the late 1990s and 2007), which correspond to major floods of summer 1990, combined ice-jam flood years of 1996 and 1997, and the ice-jam flood of 2007, respectively. At PAD 23, average total PAC concentration during the post-1972 not flood-prone period is less than half that of the pre-1972 average when the lake was flood-prone. High total PAC concentration in the uppermost sample (2010) at PAD 23 is due mainly to increases in retene and C2- through C4 phenanthrenes/anthracenes, which are elevated in river sediments near forest fires in northern Alberta [52] and elsewhere [51], and common in sediments of the Athabasca River and its tributaries both upstream and downstream of oil sands deposits [10], [44].

At PAD 18, total PAC concentration shows a gradually declining trend throughout the sediment core, except for an increase in the uppermost sample to values more typical of the late 1700s (Fig. 8). Consistently lowest total PAC concentrations occurred ∼1975–1995, a result that indicates oil sands activities are not elevating sedimentary concentrations of atmospherically-deposited PACs in the delta. Although total PAC concentration is elevated in the uppermost sample (2010), PAC composition is dominated by the parent compounds fluorene (F), fluoranthene (Fl), naphthalene (N), phenanthrene (P), perylene (PLY), and other PACs that do not have a unique association with bitumen or oil sands production (C1-biphenyls, C1- to C4 naphthalenes (N1–N4); Fig. 5, 8) [10], [44]. Oil sands-associated PACs and retene (a marker of conifer fire; [51], [52]) do not rise in the uppermost sample, which suggests oil sands development and fire cannot be the underlying causes. Instead, the rapid down-core decline in concentration of most parent PACs between the uppermost two sediment samples suggests that degradation by biological, chemical or physical processes (i.e., diagenesis) could account for the rise in the uppermost sample [46]. Consistent with this interpretation, several compounds only attain values above the detection limits in the uppermost core sample from PAD 18 (perylene, fluorene, fluoranthene, C1-biphenyls, C4-naphthalenes).

## Discussion

Sediment cores of three lakes in the PAD provide distinctly different profiles of PAC deposition over the past >200 years. Prior paleohydrological reconstructions of the study lakes were required to inform accurate interpretations of the PAC stratigraphic records. Without paleohydrological knowledge, entirely conflicting conclusions could readily have been drawn from the PAD 31 and PAD 23 records, whose profiles show increasing and decreasing supply of river-transported bitumen-associated indicator PACs during the past few decades, respectively (Fig. 7). These trends clearly reflect natural and engineered geomorphic changes in the flow of the Athabasca River [27]. Results from these two sites identify that flooding from the Athabasca River is an important vector that naturally supplies bitumen-sourced PACs to the Athabasca Delta.

Predictable responses of sedimentary PAC composition and concentration to changing hydrological conditions confirm that the sources and trends of these contaminants can be determined effectively from analyses of sediment cores from lakes in the PAD, an approach recommended by the Federal Expert Oil Sands Advisory Panel to assess environmental impacts of oil sands development [3] and proposed for the new federal Phase 2 monitoring program [21]. Here, we demonstrate that these methods yield informative results, but that detailed knowledge of hydrological variations is essential because shifting hydrological conditions at the study lakes complicates blind comparison of sedimentary PAC characteristics between stratigraphic intervals representing pre- and post-1967 development of the oil sands. Consequently, the PAC record from PAD 23 cannot be used to evaluate whether additional industrial loading of PACs has occurred, because the lake has received river floodwaters less frequently during the period since oil sands development than it did pre-development. The stratigraphic record at PAD 23 does show, however, that natural river processes, in particular ice-jam floods, have been an important source of PAC delivery to the lake in the recent past. In contrast, the increase in flood frequency at PAD 31 that has occurred since the Embarras Breakthrough in 1982 can be used to evaluate the role of industrial contributions of PACs by comparing results from this stratigraphic interval to the pre-1940 frequently-flooded interval (Fig. 5a, 7). This comparison identifies that natural processes responsible for delivering bitumen from along the banks of the Athabasca River and its tributaries can account for the PACs most associated with a bitumen origin in the post-1982 sediments of PAD 31. Thus, despite rapid growth of oil sands development during the past 25 years [1], the data reveal no measurable increase in concentration or proportion of river-transported bitumen-associated indicator PACs.

Although we fully recognize that our conclusions of no post-industrial increase in the sedimentary composition and concentration of bitumen-associated and river-transported PACs is based on only one lake record, PAD 31 is pivotally located at the upstream front of the Athabasca Delta where river floodwaters spread out over the delta [23], [26], [27]. PAD 31 is one of the most frequently flooded restricted-drainage lake in the Athabasca Delta [22], [27], and it is easily flooded both during high-water events in the spring due to snowmelt and ice-jams and during rain events in summer [27], [60]. The spring melt period and rain events have been identified as particularly important times when pulses of contaminants from oil sands development become transported to the Athabasca River and its distributaries and dispersed downstream [10], [11], [15], [20]. Thus, floodplain lake sediments can be expected to capture signals of industrial contamination. For this reason, PAD 31 should be a particularly sensitive recorder of changes in river-borne PAC transport to the numerous restricted-drainage lakes of the Peace-Athabasca Delta, and is well positioned for continued monitoring of bitumen-derived PACs from flooding of the Athabasca River.

Conclusions we have drawn from the sediment record at PAD 31 are consistent with a previous study [12] showing that concentrations of most PACs were similar in sediments deposited during the 1950s (i.e., pre-development) and the late 1990s in Lake Athabasca and Richardson Lake, lakes that continuously receive Athabasca River water. From data at these two time points, Evans *et al*. [12] concluded that there was little evidence of a temporal increase in PAC concentrations due to the oil sands industry. Moreover, concentrations of the compounds we have identified as indicative of transport by the river (D, FLPY, BAC) are similar in sediments from the PAD 31 post-1982 strata (deposited during periodic flood events) and sediments in Richardson Lake from the 1950s and late 1990s [12] (deposited during continuous inflow from the Athabasca River). Agreement of findings at these sites suggests that high-flow (flood) events on the Athabasca River are a major vector for PAC-transport to the delta, as occurs downstream in the lower Mackenzie River basin [18], [55]. This conclusion is also supported by equivalent PAC concentrations in flood deposits of PAD 31 and the upper range of concentrations in Athabasca River sediment measured by RAMP and reported in [15] (see Fig. 6). Given the importance of natural processes and industrial sources during river flood stages, monitoring and research programs must begin to sample at locations and times that capture contaminant dispersal during flood events. We propose that a network consisting of a larger number of monitoring sites and broader range of hydrological basin types is critically needed to further evaluate spatial and temporal distributions of PACs in the delta, their sources and their potential toxicity to biota. Such a monitoring network would serve as an early warning signal for any future changes in PAC delivery via the Athabasca River.

Results also reveal no evidence that industrial activity has contributed measurably to the sedimentary concentration of PACs supplied by long-range atmospheric transport and deposition in the vicinity of the PAD, as was also found for key metals of concern [61]. Dominance of PACs supplied by atmospheric transport in sediments from PAD 18 (Fig. 5) reflects pyrogenic-sourced PACs typically produced by forest fires [52], [53], and the total PAC profile (Fig. 8) indicates that this has been a decreasing source of PACs since the mid-1700s. Maximum pyrogenic-sourced PACs during the 1700s at PAD 18 may reflect more frequent or larger fires during the regionally arid climatic conditions that prevailed in elevated regions of the PAD during the Little Ice Age [23], [26], prior to European settlement and associated forest clearance and fire management. These baseline data can be used for continued monitoring of sediment PAC composition and concentration at PAD 18 to track changes in atmospheric PAC deposition to the delta as oil sands development expands, climate fluctuates and land use changes.

Our findings add new dimensions to conclusions developed by recent studies that have assessed oil sands industrial contributions to downstream PAC fluxes [10], [15]. For example, Kelly *et al*. [10] identified that, during the non-breakup period, *dissolved* PAC concentrations at sites in the Athabasca River adjacent to and immediately downstream of the oil sands mining area were significantly greater than concentrations at upstream sites. However, because PACs are strongly hydrophobic, PAC loads to the Athabasca Delta should be dominated by the *particulate* fraction that is archived in the sediments of floodplain lakes. The study by Timoney and Lee [15] did attempt to track changes in particulate PAC loads to the Athabasca Delta via use of total PAC concentration data in river-bottom sediment samples collected by RAMP during 1999–2009. Yet, this sampling design has been identified as problematic [3], [17], in part because of the shifting nature of fluvial and fluvio-deltaic sedimentary environments [16]. Our experimental methods, on the other hand, offer an alternative approach to address concerns related to the possible effects of oil sands industrial activities. An important advantage is that analyses of floodplain lake sediment profiles provide the hydrological and temporal context required to define pre-development baseline conditions and decipher trends over time due to natural versus anthropogenic processes.

As we have demonstrated, the unique perspectives offered by the lake sediment core records indicates that the PAD has been a natural repository of PAC-laden sediment carried by the Athabasca River for the past >200 years (and likely millennia). This finding is consistent with an independent estimate of natural hydrocarbon inputs from exposed bitumen-rich sediment along the Athabasca River and its distributaries between Fort McMurray and Embarras [62]. Of the estimated 6.35 Mt of suspended sediment that pass the Embarras gauging station annually, they concluded that at most 3%, and possibly 1% or less, was likely derived from the natural oil sands exposures. Assuming that 1% of the suspended sediment is derived from the natural oil sands exposures (63,500 tonnes/year), which comprise 8–14% bitumen (as reported in [4]), then natural processes may account for ∼5,000 to 9,000 tonnes/year of bitumen to the downstream delta and Lake Athabasca. By mass, this is approximately equivalent to an Exxon Valdez crude oil spill [63] deposited every four to eight years by natural processes. Using estimates of the PAC content of the oil sands sediment (0.0095% PAC by mass; from [10]) and sediments from the Athabasca River and two tributaries downstream of riverbank deposits of bitumen in areas unaffected by industrial development (0.0275% PACs by mass; from [44]), this translates to 6 - 17.5 tonnes of total PACs potentially supplied to downstream ecosystems annually by natural erosion and transport of oil sands material by the Athabasca River and tributaries. Although these estimates of natural loads of bitumen and PACs may appear high, they are diluted by ‘cleaner’ sediment from upstream locations in the watershed [62]. Consequently, the mean PAC content of sediments deposited during flood-prone periods at PAD 23 and PAD 31 within the Athabasca Delta (1.55 mg PACs/kg sediment ±0.51 (1 SD, range: 0.45–3.01 mg/kg)) is lower than that commonly measured in soils from urban environments in the USA [64], [65].

As we demonstrate in this and other studies [24], ice-jam floods are associated with peak concentrations and proportions of river-transported bitumen-associated PACs both prior to and since onset of oil sands development. This occurs because moving river-ice and high river flow velocities can mobilize and transport naturally occurring bitumen-rich materials in the riverbanks and in localized depositional accumulations downstream to the delta [62]. Similarly, studies at the Mackenzie Delta have identified that naturally occurring petrogenic PACs eroded from the mainstem of the Mackenzie River are carried with the sediment loads during the spring freshet [18] and that concentrations of PACs in floodplain-lake sediments are positively related to flood frequency [55].

Ironically, flood events, which have long been considered a crucial natural process for maintaining hydrological and ecological integrity of the delta [66] are also a major vector supplying organic contaminants. Consequently, our findings identify that the design of monitoring and research studies must adequately capture contaminant transport and deposition during the influential spring freshet and other periods of river flood stage in order to account for the substantial natural PAC loads to the Athabasca River and its distributaries, as well to capture loading by short-lived hydrological events associated with potential pulses of industrial contaminants [10], [11], [20]. Additional lakes should be cored and analyzed to verify whether our findings are representative of other parts of the delta and upstream along the Athabasca River floodplain, and to provide further critically-needed baseline information of PAC deposition over space and time. An important future research direction is to examine the toxicity of sediments throughout the delta to aquatic biota.

### Limitations

We carefully considered multiple ways to present the sedimentary data generated by our study, including in flux units (e.g., as mass of PACs per unit area per unit time) to directly assess if industrial activity is accelerating the rate of delivery of PACs to lakes in the delta. But, this was not possible based on the experimental design of this study, because at PAD 31 sedimentation rates increased since the lake became more susceptible to flooding following the Embarras Breakthrough (Fig. 2; [27]). As a consequence, it is not possible to distinguish natural hydrological causes of changes in PAC *fluxes* from those potentially due to erosion and other processes by industrial activities because they are confounded in time. Rather, we contend that if industry has substantially enhanced PAC loads to the delta, then the likelihood of doing so with the same sedimentary *concentration* and *composition* of PACs that has occurred naturally due to erosion of the Athabasca River would seem highly unlikely, but remains to be tested at additional sites. We note that Timoney and Lee [15] have concluded that oil sands industrial activity may be related to increasing sedimentary PAC *concentration* in the Athabasca River, but this was not evident in the sediment core records we generated.

The conclusions arising from this study are based on analyses performed at three lakes. While these lakes span a broad range of basin hydrology and river connectivity, they do not cover the full range that exists in the Peace-Athabasca Delta. For example, our study did not include continuously flooded open-drainage lakes due to challenges of obtaining interpretable records from systems with such temporally variable hydrological and sedimentary environments [e.g., 27]. Nor did it include lakes flooded mainly by the Peace River which traverses undeveloped surficial deposits of bitumen. Sediments from the study lakes capture PACs transported by the Athabasca River to the delta during flood stages in spring and summer, but may not record PACs dispersed during lower river stages and by the Peace River. Thus, we recommend that periodic synoptic surveys of surface sediments and water isotope tracers be conducted for a larger suite of delta lakes (40 or more; e.g., [25], [32]) that span more complete gradients of basin hydrology (i.e., lakes with open- to closed-drainage hydrology; lakes receiving inflow from the Peace River as well as the Athabasca River) to quantify relationships between connectivity with the rivers and the sedimentary concentrations and composition of PACs, and to further elucidate the potential role of Alberta’s oil sands industry on contaminant delivery to the delta. Toxicity tests performed with the surficial sediments could provide important information to examine their effects on aquatic biota in a risk-assessment framework.
